# Danhong injection in the treatment of chronic stable angina: study protocol for a randomized controlled trial

**DOI:** 10.1186/s13063-015-0998-1

**Published:** 2015-10-21

**Authors:** Peng Qian Wang, Dan Dan Li, Wei Dong, Jun Liu, Ya Nan Yu, Chun Ti Shen, Qi Guang Chen, Bing Wei Chen, Yun Dai Chen, Zhong Wang

**Affiliations:** Institute of Basic Research in Clinical Medicine, China Academy of Chinese Medical Sciences, No. 16 Nanxiaojie, Dongzhimen nei, Beijing, 100700 China; Department of Cardiology, PLA General Hospital, Beijing, 100853 China; Changzhou Traditional Chinese Medicine Hospital, Heping North Road, Changzhou, 213004 Jiangsu China; School of Public Health, Southeast University, Dijia Qiao 87, Nanjing, 210009 Jiangsu China

**Keywords:** Chinese medicine, Chronic stable angina, Danhong injection, Randomized controlled trial

## Abstract

**Background:**

Chronic stable angina is a leading cause of death worldwide. Danhong injection, a complementary alternative medicine for chronic stable angina, has been demonstrated to be effective in numerous studies and is widely prescribed to patients. However, the methodological quality of most prior studies was found to be, in general, low. Therefore, we designed this randomized controlled trial to evaluate the efficacy and safety of using Danhong injection to treat chronic stable angina.

**Methods/design:**

This is a randomized multicentre, double-blind, placebo-controlled, adaptive clinical trial. A total of 870 patients meeting the eligibility criteria will be randomly assigned into either the Danhong injection or the placebo group in a 2:1 ratio. Participants will then undergo a 2-week treatment regimen and a 76-day follow-up period. Because this is an adaptive trial, two interim analyses are prospectively planned. These will be performed after one-third and two-thirds of the patients, respectively, have completed the trial. Based on the results of these interim analyses, a data monitoring committee will determine how to modify aspects of the study without undermining the validity and integrity of the trial. The primary outcome measure is the proportion of patients who show a clinically significant change, which is defined as at least a 20-point improvement in angina frequency score on the Seattle Angina Questionnaire, which will be administered on day 30. Other secondary efficacy and safety outcomes will also be assessed.

**Discussion:**

This trial will provide high-quality evidence regarding the use of Danhong injection to treat chronic stable angina.

**Trial registration:**

ClinicalTrials.gov: NCT01681316.

## Background

Stable ischaemic heart disease is a leading cause of death worldwide, and is associated with a substantial and annually increasing burden on individuals and society [1, 2]. In the USA, the overall rate of death attributable to cardiovascular disease in 2010 was 235.5 per 100,000, and the total number of inpatient cardiovascular operations and procedures increased by 28 %, from 5,939,000 in 2000 to 7,588,000 in 2010 [1]. As one of the most common types of stable ischaemic heart disease, chronic stable angina is characterized by recurrent chest pain or pressure, known as angina, which is exacerbated by activity or stress [3]. Despite the continued exploration of myocardial revascularization procedures, preventive therapies, including lifestyle interventions, risk factor modification, antiplatelet therapy [4], renin-angiotensin-aldosterone blocker therapy, and newer anti-anginal drugs [5], some patients still experience cardiovascular events, and the proportion of patients whose quality of life is impaired by the frequent symptoms of this condition remains high [6–8]. This is thought to be associated with the undesirable side-effects of anti-anginal therapies, which include secondary nitrate lipid lapse, aspirin resistance [9] and coronary microvascular dysfunction [10].

Therefore, it is of the utmost importance to investigate the role of complementary and alternative medicines in the treatment of angina pectoris. Indeed, 64 % of patients with cardiovascular disease who were surveyed in the USA tended to use complementary and alternative medicines [11, 12], including Chinese medicines. Chinese medicines have been used in therapeutic approaches in East Asia for over two millennia. In China, Chinese patent medicines are prevalent and are commonly used as an alternative to Western medicine [13, 14]. Evidence has accumulated regarding the efficacy of Chinese medicines in treating angina pectoris [9, 15–17]. As a Chinese patent medicine, Danhong injection, which is extracted from Danshen (*Radix Salviae miltiorrhizae*) and Honghua (*Flos carthami*), is widely prescribed to patients with coronary heart disease. Danhong injection, the profile of which has been analyzed using HPLC, is certified as a Chinese medicinal product by the China Food and Drug Administration. A number of previous studies, from the bench to the bedside, have suggested that the pharmacological mechanisms of Danhong injection might involve anti-inflammatory activity, anti-atherosclerotic activity, and the activation of blood circulation [18–22]. Several clinical studies have also demonstrated that Danhong injection might be an effective and safe treatment option for the management of coronary heart disease [18, 19].

However, the methodological quality of most previous studies was assessed to be, in general, low. No critically appraised evidence, such as a well-designed randomized controlled trial, is available to provide a high level of evidence to justify the clinical use and recommendation of Danhong injection. In this study, we will conduct a randomized, multicentre, double-blind, placebo-controlled trial to investigate the efficacy and safety of Danhong injection in patients with chronic stable angina. This trial was registered (NCT01681316) at ClinicalTrials.gov.

## Methods/design

### Trial design

**Fig. 1:**
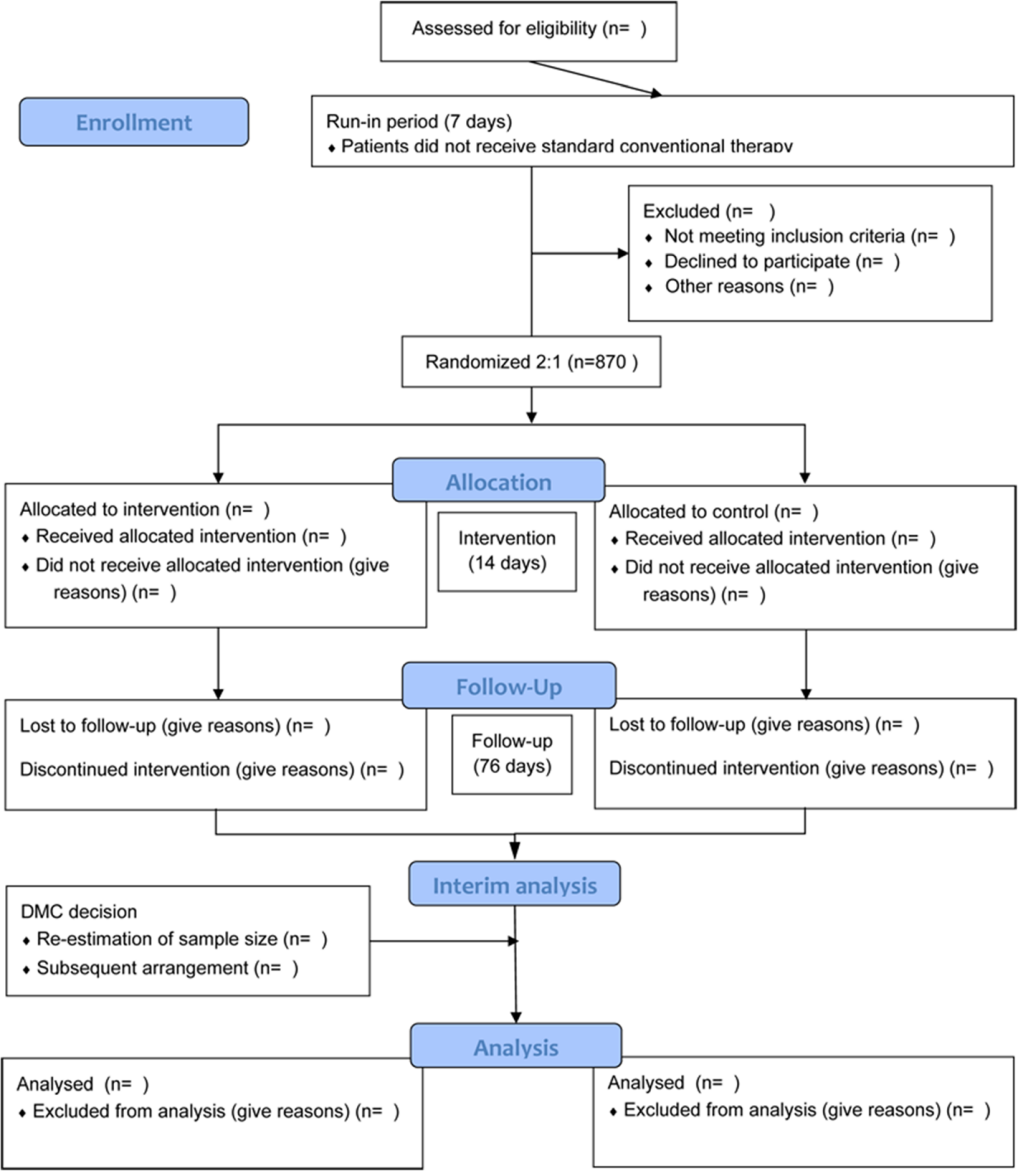
Trial flow chart: DMC, data monitoring committee

This study is an adaptive-design, randomized, multicentre, double-blind, placebo-controlled trial. Patients who meet the eligibility criteria for chronic stable angina will be randomized into either a Danhong injection or a placebo group in a 2:1 ratio (Fig. 1, Table 1).

**Table 1:** Trial process chart

Period	Run-in period	Treatment period			Follow-up period		
Day	−7	0	7	14	30	60	90
Informed consent	×	×					
Inclusion or exclusion criteria	×	×					
Medical history	×	×					
Medical examination	×						
Combined disease treatment	×	×	×	×	×	×	×
Outcome measures:							
Proportion of patients who have a clinically significant change as defined by Seattle Angina Questionnaire: angina frequency score		×			×	×	×
Symptom questionnaire of traditional Chinese medicine		×	×	×	×	×	×
The other four Seattle Angina Questionnaire scales		×	×	×	×	×	×
Frequency of anginal attack per week	×	×	×	×	×	×	×
Canadian Cardiovascular Society grade		×	×	×	×	×	×
Consumption of short-acting nitrates	×	×	×	×	×	×	×
Electrocardiogram		×	×	×	×	×	×
Serum lipids		×		×			
High-sensitivity C-reactive protein		×		×			
Platelet aggregation rate		×		×			
New-onset major vascular events							×
Overall mortality							×
Incidence of severe haemorrhage							×
Incidence of moderate haemorrhage							×
Adverse and serious adverse events			×	×	×	×	×
Exercise tolerance test (if necessary after 1st interim analysis)		×		×			
Profiles of micro-RNA in 60 patients in certain centres		×		×			×
Profiles of mRNA in 60 patients in certain centres		×		×			×

### Eligibility criteria

Eligible patients are those who meet all of the following inclusion criteria and who do not have any of the listed exclusion criteria.

#### Diagnostic criteria

1. The diagnostic criteria for chronic stable angina were determined according to the Chinese Guidelines for the Diagnosis and Treatment of Chronic Stable Angina (2007) [23], the ACC/AHA Guideline Update for the Management of Patients With Chronic Stable Angina (2002) [24], and the European Society of Cardiology Guidelines for the Management of Stable Angina Pectoris [25].
2. Traditional Chinese medicine diagnostic criteria were determined according to the Guidelines for Clinical Research of New Drugs of Traditional Chinese Medicine (2002) [26].

#### Inclusion criteria

1. Female or male inpatients.
2. Age: 18–70 years.
3. Patients with a clinical diagnosis of chronic stable angina. Patients must fulfil one of the following conditions: (a) a history of myocardial infarction and ST-T changes, (b) stenosis of more than 50 % in at least one major epicardial coronary artery, as shown by coronary angiography or computed tomography angiography, or (c) coronary heart disease confirmed by radionuclide angiocardiography.
4. Patients with a clinical diagnosis of ‘*Xueyu Zheng*’ (blood stasis syndrome), which is defined as a score of at least 15 on the Chinese Medicine Symptom Scale of ‘*Xueyu Zheng*’ for angina patients. The Chinese Medicine Symptom Scale of ‘*Xueyu Zheng*’ includes the following items: (a) chest pain (0–10); (b) chest distress (0–10); (c) palpitation (0–5); (d) purple or dark lips (0–5); (e) purple or dark tongue (0–5); and (f) unsmooth pulse (0–5).
5. Patients with moderate angina pectoris, which is defined as Grade II or III on the Canadian Cardiovascular Society Angina Grading Scale.
6. Patient is willing to voluntarily participate and to sign a written informed consent document.

#### Exclusion criteria

1. Women who are pregnant or lactating or who have a positive pregnancy test, or women who have a menstrual period at baseline.
2. Women with childbearing potential who disagree with using contraception during the treatment period.
3. Patients with severe complications that would complicate the condition, as assessed by the investigator, including liver or renal dysfunction, severe cardiopulmonary dysfunction, pulmonary hypertension, chronic obstructive pulmonary disease, a history of epilepsy or cerebral haemorrhage.
4. Patients who were angina-free during the run-in period without taking any drug.
5. Patients who experienced myocardial infarction or who were classified as Grade IV on the Canadian Cardiovascular Society Angina Grading Scale within the preceding 3 months.
6. Patients with chest pain that is caused by any other disease (e.g., acute myocardial infarction, severe neurosis, menopausal syndrome or hyperthyroidism).
7. Patients with a history of drug-induced bleeding or a history of bleeding caused by warfarin.
8. Patients with a history of haematopoietic disorder.
9. Patients who have had surgery within the previous 4 weeks or who have a haemorrhagic tendency.
10.  Patients who are participating in other trials or who have participated in other trials within the past 3 months.
11.  Patients with a history of allergy or with a known or suspected allergy to the study drug.
12.  Patients with a known or suspected history of alcohol or drug abuse within the past 2 years.
13.  Patients with a mental disorder.
14.  Patients who are unable to participate in the study, as judged by the investigator.
15.  Family members or relatives of the study centre staff.

### Recruitment

Recruitment began in December 2012 and is expected to end in December 2015. Patients with chronic stable angina will be screened according to the inclusion and exclusion criteria, to identify eligible participants. The patients will be thoroughly informed regarding the details of the study and its potential benefits and risks. Participants will be included only if they meet the inclusion criteria and willingly agree to provide written informed consent. This study is being conducted at dozens of hospitals across China.

### Sample size calculation

According to previous studies [27–32], the proportion of patients who experienced a clinically significant change that was defined as at least a 20-point improvement in a score on the Seattle Angina Questionnaire angina frequency was 30 % after one month of standard conventional therapy. In this trial, we adopted an adaptive design, which involved using the statistical sample size calculation software EAST5.2. It is hypothesized that an increase of at least 10 % is clinically significant for the Danhong injection group; therefore, the number of subjects required is initially estimated to be 726 (one-sided test, *α* = 0.05, *β* = 0.15). To allow for a 20 % dropout rate, a total of 870 patients will therefore be recruited. Because patients will be randomized into the Danhong injection group or the control group in a ratio of 2:1, the number of participants in the Danhong injection group will be 582 and the number in the control group will be 288. According to the adaptive design, the sample size can be adjusted based on the results of two interim analyses, which will be performed after one-third (288) and two-thirds (582) of the patients have completed the trial.

### Randomization

All eligible patients who consent to participation will be randomized into either the Danhong injection or the placebo group in a 2:1 ratio. Randomization will be conducted using a clinical information management system (Brightech, Somerset, USA). This system automatically randomizes patients and generates a randomization number with a message noting their assigned treatment. In addition, randomization will be stratified based on whether a patient received standard conventional therapy for more than 1 week prior to study initiation.

### Blinding

Participants and research personnel will be blinded to Danhong injection therapy or placebo treatment group assignments until the study has concluded. Because the colour of Danhong injection and 0.9 % saline are different, the dropping bottles will be wrapped in sealed shaded bags, and brown infusion devices will be used for infusion. These procedures will be implemented by two professional nurses who will be required to sign a confidentiality agreement before study initiation and not to contact each other. One of the professional nurses will be in charge of preparing the drugs in a special transfusion room and sealing the infusion bottles with shaded brown bags. The other nurse will take the prepared drugs from the transfusion room to the infusion nurse and supervise the infusion process to ensure that the allocation of the drugs is blinded to the patients (the shaded brown bags will not be unwrapped during infusion and will be checked for integrity after infusion).

### Interventions

Standard conventional therapy will be provided to all of the included participants throughout the trial, in strict accordance with the Chinese Guidelines for the Diagnosis and Treatment of Chronic Stable Angina (2007) [23]. Standard conventional therapy includes:

-  Antiplatelet agents: aspirin (75–100 mg, once per day) or clopidogrel (if the patient is intolerant to aspirin). Patients with a history of percutaneous coronary intervention will be prescribed both of these agents.
-  Lipid-lowering agents (statins): atorvastatin (10–20 mg, once per day) or simvastatin (20–40 mg, once per day).
-  Anti-angina agents: *β*-blockers (metoprolol 50–200 mg, once per day, or analogous agents); long-acting nitrates (isosorbide mononitrate 40–60 mg, once per day); or calcium channel blockers (amlodipine 5–20 mg, once per day).
-  Patients with diabetes and hypertension will be advised to take angiotensin-converting enzyme inhibitors or angiotensin-receptor blockers (e.g., lisinopril 10–20 mg, once per day, or losartan 50 mg, once per day) as a secondary preventive measure for chronic stable angina.

All of these basic treatments will be recorded in detail in the patients’ medical records as well as in their case report forms.

Participants in the Danhong injection group will be treated using Danhong injection (40 ml per day) plus 0.9 % normal saline (250 ml intravenously per day), while participants in the placebo group will be treated using placebo (0.9 % normal saline, 40 ml per day) plus 0.9 % normal saline (250 ml intravenously per day). Participants who received standard conventional therapy for more than 1 week prior to study initiation will be randomized directly into one of the groups; otherwise, a run-in period of 1 week of standard conventional therapy will be performed before randomization. All of the included patients will undergo a 2-week treatment regimen and a 76-day follow-up period.

Patients will be instructed to take a nitroglycerin tablet (0.5 mg per tablet, provided by Beijing Yimin Pharmaceutical Co., Ltd.) in case of an angina attack, and this dose can be repeated approximately every 5 minutes until the angina is relieved. If the angina persists after three doses, the patient should be transported to hospital immediately for further medical treatment. Patients will be asked to record the details of administration times, the number of doses and the dosages of nitroglycerin in a patient diary, which will be collected by the investigators at a subsequent study visit.

Concomitant medications that are deemed necessary to manage any serious underlying disease will be allowed during the study and should be recorded in a patient’s medical records and case report forms. However, participants are not allowed to take any other Chinese herbal medicines or Chinese patent medicines that have the effect of promoting blood circulation or removing blood stasis throughout the study period.

### Quality control for the intervention

All of the staff, including the operators, investigators, data collectors and analyzers, are to be strictly trained. In each clinical centre, special transfusion rooms should be established and staffed with professional nurses. For outpatient appointments, physicians (investigators) must fill out medical record cards and medications record cards in a timely manner.

To abide by the blinding principle, the placebo operation must be performed by two professional nurses who were required to sign a confidentiality agreement to commit to not having contact with each other. One of the professional nurses will be in charge of drug preparation and will never be allowed to have contact with the investigators, analyzers, sponsors or organizers of the trial throughout the trial, and the other will be responsible for sending prepared drugs to patients.

### Outcome measurements

The primary outcome is the proportion of patients with a clinically significant change, which is defined as showing at least a 20-point improvement on the Seattle Angina Questionnaire angina frequency score on day 30. The Seattle Angina Questionnaire is a 19-item questionnaire that quantifies physical limitations due to angina, any recent change in the severity of angina, the frequency of angina, satisfaction with treatment and quality of life. The total score can range from 0 to 100, and higher scores indicate a better health status [31].

The secondary outcome measures include:

- The total score of symptoms in a questionnaire related to traditional Chinese medicine;
- The proportion of patients who experience clinically significant changes in the other four Seattle Angina Questionnaire domains;
- The frequency of anginal attacks per week;
- Angina grade, according to the Canadian Cardiovascular Society Angina Grading Scale;
- Consumption of short-acting nitrates;
- Changes in electrocardiogram results;
- Changes in serum lipid levels, high-sensitivity C-reactive protein levels and the rate of platelet aggregation.

The safety of Danhong injection will be evaluated by the incidence of new-onset major vascular events within 90 days, the overall mortality within 90 days, the incidence of severe haemorrhages within 90 days, the incidence of moderate haemorrhages within 90 days, and the incidence of adverse and serious adverse events.

In addition, other pre-specified outcome measures, including changes from baseline in an exercise tolerance test performed on day 14 in 290 patients for the first interim analysis and changes in the micro-RNA and mRNA profiles of 60 patients selected at the Chinese PLA General Hospital and Xuanwu Hospital Capital Medical University. An overview of the measurements collected for data is shown in Table 1.

After each interim analysis, the data monitoring committee will determine whether it is necessary to continue, modify or terminate the collection of these outcome data.

### Safety and adverse events

An adverse event is defined as any unexpected and uncomfortable medical occurrence in a patient or clinical investigation subject being administered a pharmaceutical product. The event does not necessarily have to have a causal relationship with the study treatment.

A serious adverse event is defined as any adverse event that fulfils at least one of the following criteria:

- Is fatal;
- Is a life-threatening event;
- Leads to inpatient hospitalization or the prolongation of existing hospitalization;
- Leads to persistent or significant disability or incapacity;
- Leads to a congenital anomaly or birth defect;
- Is medically significant or requires intervention to prevent one or more of these outcomes.

All adverse events that occur in the clinical study must be recorded on the case report form and must include the following contents: type, intensity, emergence time, duration, action taken and process. The relationship between the adverse event and the studied drug must be assessed and recorded by the investigator, in consideration of complications and the combined use of drugs.

If adverse events occur, the investigator will determine whether the participant should withdraw from the study, according to the condition of the patient, and follow-up procedures will be performed and recorded in detail. Safety issues will also be reviewed and combined with incidence data and data concerning the treatment of the patient, to determine whether the adverse events are associated with the investigated drug.

For serious adverse events, the investigator must immediately take necessary measures, report to the principal investigator and the ethics committee, and place a record on the case report form including a signature and the date. The classification of the severity of adverse events and their relationship to the studied drug will be conducted according to the methods in our previous study [33].

### Interim analysis

Because this is an adaptive trial, two interim analyses are prospectively planned, and these will be performed in a blinded manner after one-third and two-thirds of the patients, respectively, have completed the trial. The statistical results of the interim analyses will be relayed to the data monitoring committee, who will decide on the re-estimation of sample size and determine whether any subsequent modifications must be made to the trial protocol. Because the two interim analyses may lead to an increased possibility of a type I error, we will apply the Lan–DeMets alpha spending function with an O’Brien–Fleming boundary to adjust the results.

### Data collection and management

This trial is divided into three phases: a run-in period, a treatment period and a follow-up period. An overview of the study visits and data collection is provided in Table 1.

All data will be recorded by trained clinical investigators using standardized electronic case report forms and Brightech clinical information management systems (Brightech, Somerset, NJ, USA). The accuracy and reliability of the data will be ensured by the study monitor, who will verify and cross-check the electronic case report forms against the investigator’s records (source document verification), using the maintenance of a drug-dispensing log by the investigator. Front-end checks in the electronic case report forms and back-end checks in the clinical information management system will be used as a validation check of the data. If there are any discrepancies in the electronic case report forms, the results will be sent to the investigator for resolution. To facilitate analysis of the data, the treatment code will be released to the statisticians. The results of the analysis must not be released with individual identification of the subjects until the database is closed.

To ensure the effectiveness and integrity of the trial design, three committees have been established by the multicentre trial coordination group: the clinical trial guidance committee, the data monitoring committee (also known as the data and safety monitoring board) and the outcome evaluation committee. The first committee is responsible for the study design and the implementation process. The data monitoring committee will supervise the integrity and accuracy of data collection to control its quality. The outcome evaluation committee will evaluate key outcomes (including outcome measurements and adverse events) based on their clinical expertise.

### Statistical analysis

Statistical analysis will be performed using Statistical Analysis System version 9.1 (SAS Institute Inc, Cary, NC, USA) statistical software packages. Most outcome measurements (except the micro-RNA and mRNA profiles) will be analyzed using full analysis sets and per-protocol sets according to intention-to-treat analysis. Safety analysis will be performed in a safety set, which is defined as a subset of subjects who were randomized and who received at least one treatment.

The statistical analysis will include the distribution of subjects, baseline characteristics of participants, compliance and concomitant medication, efficacy analysis and safety analysis. For parametric tests, we will describe the results using the mean (standard deviation), maximum, minimum, median and non-parametric test median (quartile deviation). Categorical data will be described as absolute values and proportions.

Quantitative data will be compared using analysis of variance (ANOVA) and *t* tests. Paired *t* tests will be used to analyze significant differences between pre- and post-treatment time points. Two-sample *t* tests will be employed for comparisons between treatment groups. After ANOVA, we will then use Student–Newman–Keuls significance tests for pairwise comparisons. Enumeration data will be analyzed using *χ*^2^ tests, Cochran–Mantel–Haenszel *χ*^2^ tests, Fisher’s exact tests or Wilcoxon rank tests; *χ*^2^ tests will be used for nominal categorical data and rank sum tests will be used for ordinal categorical data. Analysis of covariance (ANCOVA) will be used to control potential confounding variables. Significance tests will be two-tailed, with a statistical probability of *P* <0.05.

### Distribution of subjects

Analytical statistics will be calculated to estimate the difference in the number of participants who have completed or who have been withdrawn from the trial between groups.

### Baseline characteristics

Baseline characteristics in each group will be analyzed using descriptive statistics, including means or medians for continuous variables and percentages for categorical variables.

### Compliance and concomitant medication

Compliance analysis will be based on full analysis sets, and analysis of concomitant medications will be based on safety sets.

### Efficacy analysis

Primary and secondary efficacy parameters will be analyzed. Any factors impacting efficacy, such as age and sex, should be taken into account as covariants, and an ANCOVA model, Cox’s proportional hazards regression model, or logistic regression model will be used to assess treatment effects for these factors. In addition, study participants will be classified according to whether they have been treated with long-acting nitrates and will then be enrolled into a study stratum. We will conduct stratified analysis, and each stratum will be analyzed separately.

### Safety analysis

Safety will be analyzed in terms of the incidence of new-onset major vascular events within 90 days, the overall mortality within 90 days, the incidence of severe haemorrhages within 90 days, the incidence of moderate haemorrhages within 90 days, and adverse and seriously adverse events.

### Ethics

This trial has been approved by local institutional ethics committees (the ethics committees of the Institute of Basic Clinical Research, China Academy of Chinese Medical Sciences and of Chinese PLA General Hospital). This trial will be conducted in adherence to the Declaration of Helsinki (Edinburgh 2000). Informed written consent will be required of all participants.

## Discussion

It is widely accepted that a randomized controlled trial is the gold standard for evaluating the clinical efficacy and safety of a Chinese medicine and for providing critical evidence to develop and guide treatment strategies. However, it has been suggested, controversially, that the principle of a randomized controlled trial goes against the doctrine of traditional Chinese medicine as personalized medicine. There are several drawbacks in the methodological quality of most Chinese medicine trials [34]. These include inadequate randomization, a lack of double blinding, non-placebo controls and incomplete outcome data. All of these lead to various biases that can weaken the credibility of the evidence.

There are several strengths in the methodological design and interventions described in this study. First, this is the first rigorously designed randomized controlled trial to evaluate the efficacy and safety of Danhong injection for chronic stable angina. Although a number of trials on Danhong injection have been published, there is a lack of well-designed trials that examine the efficacy of Danhong injection for the management of chronic stable angina. In this study, a central online randomization system is used for random sequence generation and allocation concealment, which might avoid potential selection bias. The blinding of both the participants and the study personnel and the blinding of outcome assessments are used to reduce the potential for performance bias and detection bias, respectively. Most previously published randomized controlled trials lack a placebo control. We understand that some critical characteristics of commonly used herbal medicines and Chinese patent medicines, such as colour, appearance, odour, and other properties, restrict the wide application of placebo controls in Chinese medicine studies. However, placebo control is strictly implemented in our study. The dosage form of an injection has a remarkable placebo effect. We employ wrapped dropping bottles and brown transfusion devices to facilitate a placebo control. Thus, the treatment appears to be identical between the Danhong injection and placebo groups. We assume that a potential placebo effect can be excluded in our study. An add-on study design is also applied in this trial, which can be summarized as an ‘A + B versus B’ model. In this model, participants are randomized to receive either intervention A combined with intervention B or intervention B alone. This method is widely used in Chinese medicine studies, where Chinese medicine is added to a conventional treatment [35]. However, the effect of Chinese medicine therapy will be exaggerated if no rigorous control is used to exclude a placebo effect. In view of this, we designed and implemented the study in a scientific, strict and cautious manner to provide reliable evidence regarding the use of Danhong injection as a complementary therapy for treating chronic stable angina. Finally, in this trial, we also adopted an adaptive design, which is defined as a study that includes a prospectively planned opportunity for the modification of one or more specified aspects of the study design and hypotheses based on the analysis of accumulating data (usually interim data) from subjects in the study [36]. This method will make the study more efficient (e.g., have a shorter duration or include fewer patients) and is more likely to demonstrate an effect of the drug if one exists [36, 37].

Second, the outcome measures in our study are patient-centred. Currently, the assessment of treatment efficacy is being transformed from a traditional doctor-reported outcome model to a patient-reported outcome model, indicating that more attention is being paid to the quality of life of the patients and the relief of symptoms. Guidelines for the management of chronic stable angina also recommend that the treatment goal should include the relief of symptoms [3]. Traditional Chinese medicine has been suggested as a promising option for alleviating symptoms and improving quality of life. Therefore, we use changes in Seattle Angina Questionnaire scores and traditional Chinese medicine syndrome scores as our outcome measures because they both focus on patient symptoms. This will help us to provide reliable and convincing evidence for the efficacy of Danhong injection.

Finally, another key point of this study is that the study drug has substantial therapeutic value in real-world clinical practice. It has been proposed that blood stasis syndrome (*Xueyu Zheng*) is the most common type of chronic stable angina [9]. A recently published review also demonstrated that Chinese patent medicines that promote blood circulation and remove blood stasis, when used as adjunctive treatments for routine anti-anginal therapy, play an active role in reducing the incidence of primary endpoint events, decreasing anginal attack rates, and improving electrocardiogram results [9]. Danhong injection is a certified Chinese medicinal product that is extracted from Danshen (*Radix Salviae miltiorrhizae*) and Honghua (*Flos carthami*). According to the basic theory of Chinese medicine, Danhong injection can produce a remarkable curative effect by promoting blood circulation and removing blood stasis. Moreover, numerous studies, both *in vivo* and *in vitro*, have validated the vascular protective effects of Danhong injection [18–22, 31, 38]. Based on the theoretical and clinical evidence mentioned previously, Danhong injection is regarded as a promising therapeutic option for the treatment of chronic stable angina.

However, there are also several limitations in this study. One of the major drawbacks lies in the lack of assessment of Danhong injection’s long-term effects on primary outcome measures. In our study, the follow-up period is only 76 days, which is relatively short. Owing to the short length of the follow-up period and limitations on our budget, the potential role of Danhong injection in reducing major vascular events and overall mortality over the long term will remain unknown. In addition, although an add-on study design and a placebo control will be applied in our study to interpret the complementary effect of Danhong injection, we will still be unable to draw a definite conclusion over whether Danhong injection can serve as an alternative therapy for chronic stable angina because we do not use a ‘head-to-head’ design. Therefore, well-designed randomized controlled trials that compare Danhong injection with conventional anti-anginal therapies and that have longer follow-up periods are needed in the future.

## Trial status

The study began recruiting patients in 2012. The trial is currently recruiting patients.

## Abbreviations

ANCOVA: analysis of covariance
ANOVA: analysis of variance
HPLC: high-performance liquid chromatography
